# 4D-Printed Redox-Responsive Needle-Flow Reactors Enabling Online Quantitative Profiling of Living Rat Brain Extracellular Lactate and Glucose

**DOI:** 10.1021/acs.analchem.5c01036

**Published:** 2025-06-24

**Authors:** Hsiao-Chu Chiu, Cheng-Kuan Su

**Affiliations:** Department of Chemistry, 34916 National Chung Hsing University, Taichung 402202, Taiwan, R.O.C.

## Abstract

Various assays are performed based on the use of oxidase-based derivatization schemes coupled with chromogenic or fluorogenic reagents and spectrometric determination. To enable online quantitative profiling of living rat brain extracellular glucose and lactate without requiring a spectrophotometer or fluorometer, a flow reactor with a redox-responsive needle is fabricated using the digital light processing four-dimensional printing (4DP) technique and photocurable resins incorporating 2,2′-(ethylenedioxy)­diethanethiol (EDT). Upon exposure to hydrogen peroxide (H_2_O_2_), oxidation of the thioether groups of the copolymer increases the polarity and swelling of the EDT-incorporated layer, inducing [H_2_O_2_]-dependent bending of the needle. Coupling this device with glucose- and lactate-oxidase-mediated reactions facilitates the reliable determination of glucose and lactate. Furthermore, the needle returns to its original state after treatment with a sodium borohydride solution, demonstrating the reversible, redox-responsive shape programming for repeated measurements. Integration of the microdialysis sampling apparatus, oxidase-based derivatization schemes, and 4D-printed redox-responsive needle-flow reactors into an online automatic analytical system achieves the method’s detection limits of 0.3 μM for glucose and 0.4 μM for lactate. To verify the reliability and applicability of this method, we perform analyses of human urine and sweat, fetal bovine serum, and rat plasma with their spike analyses, compare the results with those obtained from assay kits, and profile living rat brain extracellular glucose and lactate levels with triggered neuronal depolarization. The findings demonstrate that 4DP technologies enable the efficient fabrication of redox-responsive analytical devices and enhance the applicability of conventional enzymatic assays for online quantitative chemical analysis.

## Graphical abstract

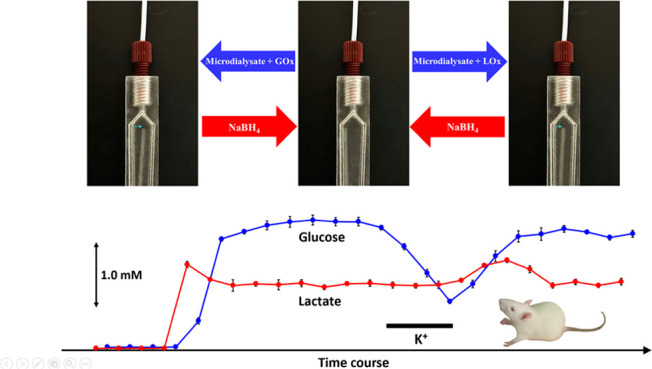

## Introduction

Probing brain extracellular glucose and lactate is essential for understanding energy metabolism related to neuronal activities and the regulation of ion homeostasis in both normal and pathological conditions.^1–5^ Implanting enzyme-based electrochemical biosensors directly into specific brain regions is a common and straightforward approach for revealing their dynamic profiles in freely moving or anesthetized animal models, owing to their small size and high temporal response.^5–8^ However, fouling of the sensing components by biological matrices and coexisting redox-active species can introduce unpredictable signal bias, which compromises the stability and reliability of implanted biosensors for long-term monitoring.^5,7–12^

Alternatively, microdialysis (MD) is an effective sampling technique that can sample extracellular molecules with a molar mass lower than the molecular weight cutoff (MWCO) of the membrane, thereby excluding most potential interferences and minimizing fouling of sensing components. This makes MD suitable for coupling with various analytical schemes to identify and calibrate sampled substances.^13–15^ When coupled with a specific oxidase-based derivatization reaction, the generated stoichiometric amount of hydrogen peroxide (H_2_O_2_), which selectively oxidizes chromogenic or fluorogenic reagents (e.g., 3,3′,5,5′-tetramethylbenzidine and 2′,7′-dichlorodihydrofluorescein) in the presence of horseradish peroxidase, enables the optical spectrometric or fluorescence-based determination of sampled extracellular glucose and lactate.^16–23^ However, several concerns remain, including the labor-intensive collection and handling of microdialysates, specialized operation of chromogenic and fluorogenic reagents, and the need for expensive spectrophotometers, fluorometers, or microplate readers. More efficient determination strategies for advancing enzymatic derivatization schemes are needed to improve the applicability of MD sampling for the online profiling of dynamic variations in brain extracellular energy metabolites.

Stimuli-responsive materials, capable of sensing environmental changes, such as temperature, humidity, light, [H^+^], and magnetic fields, are increasingly used to fabricate devices with stimuli-induced shape programming.^24–29^ For analytical applications, these devices hold potential as sensing components for quantitative chemical analysis,^27–33^ such as pH-responsive microneedles for quantifying glucose levels in interstitial fluids by measuring the changes in their heights;^31^ pH-responsive urease-immobilized circular bilayer actuators for quantitative analysis of urea in sweat samples based on their diameter;^32^ and glucose-responsive hydrogel for mechanical readouts of glucose concentrations through observing its recovery from the bending status.^33^ However, the reported stimuli-responsive devices are typically fabricated using preprepared masks or templates for stepwise stacking of stimuli-responsive and/or nonresponsive materials, followed by additional alignment, bonding, and postfabrication treatments.^31–36^ These template-assisted procedures are labor-intensive, time-consuming, and complicated, limiting the applicability of fabricated devices for analytical works. Therefore, more efficient fabrication schemes for stimuli-responsive materials are essential for developing next-generation stimuli-responsive devices capable of enabling the online profiling of brain extracellular glucose and lactate.^27–30^

Four-dimensional printing (4DP) technologies, based on conventional three-dimensional printing (3DP) of stimuli-responsive materials, effectively fabricate stimuli-responsive devices that exhibit stimuli-induced, time-dependent shape programming and geometric functions of 3D-printed devices as the fourth dimension.^37–40^ In recent years, the application of 4D-printed stimuli-responsive analytical devices has grown,^41–44^ but to the best of our knowledge, no analytical scheme that enables online quantitative chemical analysis based on the shape programming of 4D-printed devices has been reported. Therefore, new fabrication strategies and stimuli-responsive materials could enable these devices to function as universal sensing components for reducing the cost and complexity of developing analytical systems for sensitive, reliable, and high-throughput determination of glucose and lactate in rat brain microdialysates.

Inspired by the need for more efficient strategies to fabricate redox-responsive devices for the online quantitative profiling of living rat brain extracellular glucose and lactate without relying on chromogenic or fluorogenic reagents and sophisticated spectrometric determination techniques, we utilize the digital light processing 4DP technique and 2,2′-(ethylenedioxy)­diethanethiol (EDT)-incorporated photocurable resins to fabricate a flow reactor with a redox-responsive needle as the detection device.^45,46^ When an H_2_O_2_-containing sample passed through the reactor, the oxidation of the thioether groups in the copolymer increased the polarity and swelling of the EDT-incorporated layer, while no swelling occurred in the nonresponsive layer, leading to [H_2_O_2_]-dependent bending of the needle. By coupling with the glucose oxidase (GOx)-mediated oxidation of glucose and lactate oxidase (LOx)-mediated oxidation of lactate, the increased [H_2_O_2_] induced bending of the needle to allow for reliable determination of glucose and lactate in microdialysates. The needle returned to its initial state after treatment with a sodium borohydride solution,^47^ demonstrating the reversible shape-programming property. After integrating the MD sampling apparatus, oxidase-based derivatization schemes, and 4D-printed redox-responsive needle-flow reactors into an online automatic analytical system, we evaluated its analytical performance and validated its reliability and applicability by performing the analyses of human urine and sweat, fetal bovine serum, and rat plasma with their spike analyses, comparing the results with assay kit outcomes, and profiling living rat brain extracellular glucose and lactate with the neuronal depolarization triggered by perfusing a K^+^ solution through the implanted MD probe.

## Experimental Section

### Chemicals

EDT (465178), H_2_O_2_ [30% (w/w); 31642], NaBH_4_ (452882), d-(+)-glucose (G7528), GOx (G7141, from *Aspergillus niger*), l-(+)-lactic acid (L1750), LOx (L9795, from ), trisodium citrate dihydrate (C8532), disodium hydrogen phosphate (Na_2_HPO_4_; S0876), sodium chloride (NaCl; V900058), potassium chloride (KCl; P9541), glucose (GAGO20, traceable to the National Institute of Standards and Technology standard), and lactate (MAK064) assay kits were purchased from Sigma-Aldrich. Water purified using a Milli-Q IQ 7000 water purification system (Merck Millipore) was used to prepare solutions. Aqua Clear resins [Phrozen; comprising bisphenol A ethoxylate dimethacrylate, 4-acryloylmorpholine, and diphenyl­(2,4,6-trimethylbenzoyl)­phosphine oxide (as the photoinitiator)] were used to fabricate the flow reactor and the nonresponsive part of the needle, while the resins incorporating EDT were used to fabricate the redox-responsive part (Figure S1).

### 4DP of Redox-Responsive Needle-Flow Reactors

The needle-flow reactor consisted of a rectangular needle, a diamond-shaped flow cell with a groove, and a cover featuring a finger-tight adaptor that fit into the groove of the flow cell (Figures 1A and S2). The flow cell included two fittings (upper loading and bottom waste ports) for a standard 10–32 flat-bottom male connector (Figure S2A). The needle, composed of a redox-responsive (red) layer properly attached to a nonresponsive (blue) layer, was positioned at the bottom of the flow cell (Figures 1B and S2B). These components were modeled using SolidWorks 2020 (Dassault Systèmes) computer-aided design (CAD) software and fabricated via a DLP 3D printer (Phrozen Sonic Mini 8K) using a print–pause–print procedure. The .stl files of the reactor were provided in the Supporting Information. The curing time was set at 18 s with a *z*-axis resolution of 100 μm. The fabrication times were 44 min for the flow cell, 17 min for the cover, and 5 min for the needle, with corresponding weights of 9.4, 1.9, and 0.3 g, respectively. The total material cost was US$ 0.44. The flow cell and cover were fabricated using Aqua Clear resins. For the 4DP of the redox-responsive needles, the nonresponsive layer (thickness: 0.5 mm) was first fabricated using Aqua Clear resins. The resins in the vat were replaced with EDT-incorporated Aqua Clear resins to fabricate the redox-responsive layer (thickness: 0.5 mm), ensuring proper attachment to the nonresponsive layer. After fabrication, all three parts were washed with an ethanol solution. The needle was inserted into the hole at the bottom of the flow cell to align it to the initial position. The cover was then fitted, along with two flat-bottom male connectors (*p*-840 and *p*-844, IDEX Health & Science) with a short piece of polytetrafluoroethylene (PTFE) tubing (inner diameter of 0.03 in.) (Figures 1B and S2C). The assembled needle-flow reactor was washed with 10 mM citrate buffer (pH 6.0) for 2 h to remove residual resins and contaminants.

**Figure 1:**
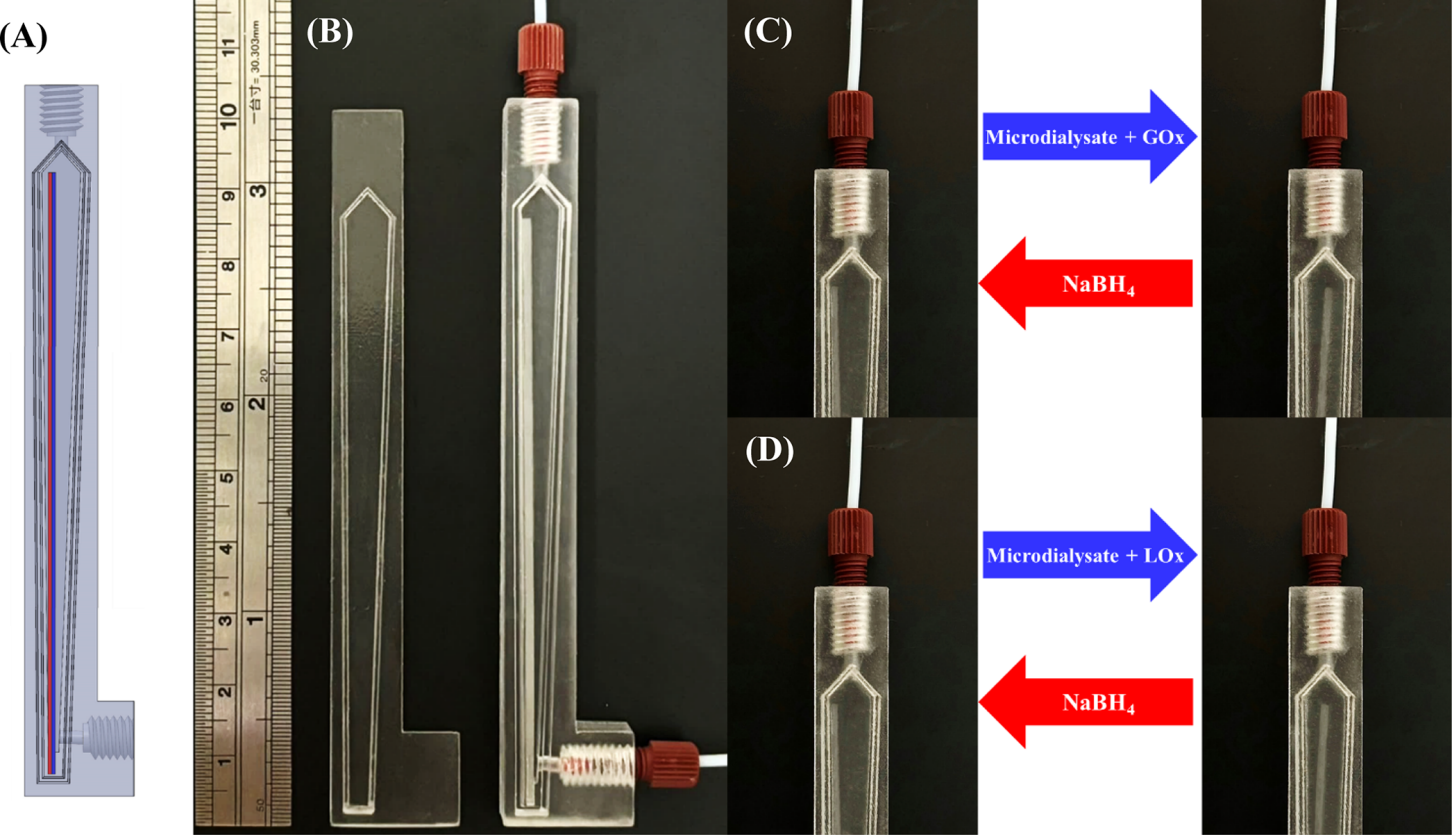
(A) CAD drawing of the needle-flow reactor featuring a redox-responsive needle, comprising a redox-responsive (red) layer attached to a nonresponsive (blue) layer, positioned at the bottom of the flow cell. (B) Photographs of the (right) diamond-shaped flow cell with the groove, redox-responsive needle, and two fittings with flat-bottom male connectors and (left) cover with the finger-tight adaptor for fitting to the groove on the flow cell. (C) The needle-flow reactor before loading of a microdialysate (left) and after loading of a GOx-treated microdialysate with a stopped-flow step to induce the bending of the needle for glucose determination (right; bending of the needle: 1.81 mm; glucose: 1.81 ± 0.04 mM). (D) The needle-flow reactor before loading of a microdialysate (left) and after loading of a LOx-treated microdialysate with a stopped-flow step to induce the bending of the needle for lactate determination (right; bending of the needle: 1.32 mm; lactate: 0.93 ± 0.02 mM).

### Methods and Apparatus

The steps of sample loading, evacuation, needle reduction, and reconditioning were required for the determination of glucose and lactate in microdialysates using 4D-printed needle-flow reactors (Figure S3). First, the microdialysate (1.0 μL min^–1^) was mixed online with a stream (9.0 μL min^–1^; 10-fold dilution) of either a GOx (1250 U mL^–1^) or an LOx (25 U mL^–1^) solution via a mixing tee (ZT1MFPK, Valco) before being loaded into two 50-μL sample loops. A carrier stream of the 10 mM citrate buffer (pH 6.0) at a flow rate of 5.0 mL min^–1^ was used to deliver the oxidase-treated microdialysates into the flow cells (volume: 1.25 mL; 25-fold dilution), with a stopped-flow step (loading time: 16 s) to stabilize the bending of the needles (step 1:5 min). To minimize spatial factors that could contribute to fluctuations in the bending of the needle, the needle-flow reactors were fixed horizontally on a digital chilling-heating dry bath (40 °C; EchoTherm, Torrey Pines Scientific). The GOx-mediated oxidation of glucose and LOx-mediated oxidation of lactate increased [H_2_O_2_] and induced the bending of the needle due to the imbalance between the oxidation-induced swelling of the EDT-incorporated and nonresponsive layers. Images of the needles were captured using a smartphone (iPhone 12) before and after bending and analyzed with ImageJ software (version 1.54d) to determine the bending of the needle, which was measured as the displacement of the left endpoint of the needle (Figure 1C,D). The measurement data were used to construct calibration curves for glucose and lactate determination. After measurement, the loaded sample was evacuated using an air stream (flow rate: 5.0 mL min^–1^; step 2:44 s). Next, a NaBH_4_ solution [2.5% (w/v) in a 1.0 mM phosphate buffer (pH 8.0)] was loaded into the flow cell (flow rate: 5.0 mL min^–1^; loading time: 16 s), followed by a stopped-flow step to restore the needle to its initial position (step 3:5 min). Then, the needle was reconditioned with the 10 mM citrate buffer (pH 6.0) before the next sample was loaded (flow rate: 5.0 mL min^–1^; step 4:44 s). As illustrated in Figure S3, the GOx-treated microdialysate was delivered into one needle-flow reactor for glucose determination, while the microdialysate was mixed online with the LOx solution and loaded into the sample loop for lactate determination in the other reactor. All steps were performed in an automated online analytical system (Figure S4 and Table S1), which was configured for parallel sample loading and measurement. The system incorporated two two-position, ten-port switching valves (C22Z–3180, Valco), two multiposition, ten-port stream selectors (C5FH-2345EMT, Valco), and two peristaltic pumps (Miniplus 3, Gilson), and was programmed using a laptop to eliminate reactive oxygen species from the laboratory environment and minimize errors from manual operation.

### Sample Preparation and Analysis of Real Samples

The method’s reliability and applicability were verified through analyses and spike analyses (spiked concentration: 1.0 mM for glucose and lactate) of human urine and sweat collected from volunteers, qualified fetal bovine serum (FBS; 10437-028, Thermo Fisher Scientific; lot number: 2185285), and rat plasma collected from a Sprague–Dawley (SD) rat. These samples were diluted with the 10 mM citrate buffer (pH 6.0; 50-fold for urine; 100-fold for sweat, serum, and plasma), filtered through 0.45-μm syringe filters (CHROMAFIL Xtra H-PTFE, Macherey-Nagel), treated with GOx or LOx, and loaded into the needle-flow reactors, which were maintained at 40 °C to measure the bending of the needle. In addition, glucose and lactate assay kits, combined with a microplate reader (Synergy H1, BioTek), were used to validate the method by comparing the results to those obtained from the 4D-printed needle-flow reactors. Statistical comparisons were performed using Student’s two-tailed unpaired *t*-test.

### Animal Experiments

An MD probe with a 500-μm diameter and a 4 mm length polyarylethersulfone membrane (MWCO: 20 kDa; 8010435; CMA Microdialysis), two plastic syringes (4606051 V, B. Braun), and a dual-channel syringe pump (KDS260, KD Scientific) operating at a perfusion flow rate of 1.0 μL min^–1^ were used to sample extracellular glucose and lactate from living rat brains, with recoveries of 26.9 ± 1.0% for glucose and 30.6 ± 0.5% for lactate. The outlets of the two syringes and the inlet of the MD probe were connected to a six-port, two-position switching valve (C22Z-3186, Valco) to switch the perfusate from 0.9% NaCl to 1.15% KCl. Male SD rats (449 ± 10 g; *n* = 12, specifically pathogen-free) from BioLASCO were acclimatized to environmentally controlled quarters (25 °C; 12-h light/12-h dark cycle) with ad libitum access to water and food. All animal treatments and experimental protocols conformed to the guidelines and approval of the Institutional Animal Care and Use Committee at National Chung Hsing University (approval number: 109-161). The rats were anesthetized with urethane (1.5 ± 0.1 g kg^–1^ body weight via intraperitoneal administration), and each rat’s head was mounted on a small-animal stereotaxic instrument (Model 900LS; David Kopf Instruments). After implanting the MD probe to target the hippocampus (2.0 mm anterior–posterior and 2.0 mm laterally from the bregma),^48^ 20 measurements (120 min) were conducted to determine the rat brain extracellular glucose and lactate concentrations upon reaching hemostasis around the insertion region. Local neuronal depolarization was then induced by switching the perfusate from 0.9% NaCl to 1.15% KCl for six measurements (36 min) before switching back for 14 measurements (84 min). Each rat was euthanized upon completion of the experiment.

## Results and Discussion

### 4DP of Redox-Responsive Needle-Flow Reactors

For the online quantitative profiling of living rat brain extracellular glucose and lactate using oxidase-based derivatization schemes rather than chromogenic or fluorometric analysis, we employed the print–pause–print DLP 4DP technique and EDT-incorporated photocurable resins to fabricate a flow reactor with a redox-responsive needle. This design enabled the reliable determination of glucose and lactate in rat brain microdialysates by measuring the induced bending of the needle (Figure 1C,D). To achieve the optimal analytical performance, we systematically optimized the composition of the photocurable resins, the design of the needle-flow reactor, the reduction conditions for the needle, the operating parameters of the online automatic analytical system, and the enzymatic derivatization conditions.

The incorporation of EDT and the design of the needle both played key roles in determining the imbalance between the redox-responsive and nonresponsive layers in the presence of H_2_O_2_ (10 μM), which, in turn, induced bending of the needle. As shown in Figure 2A, the bending of the needle increased as the concentration of incorporated EDT was raised from 2.5% to 7.5% (v/v) because of the proportional increase in the number of thioether groups in the copolymer. However, the bending decreased beyond 7.5% as the EDT-incorporated layer became softer, which reduced the imbalance between the two layers. Based on these results, we selected a concentration of incorporated EDT at 7.5% for fabricating the redox-responsive needle. Figure S5A demonstrates that the bending of the needle increased as the length was extended from 2.0 to 8.0 cm, driven by the proportional increase in the swelling-induced imbalance between the two layers. The bending decreased slightly when the length was further increased from 8.0 to 14 cm, presumably because the heavier needles counteracted the swelling-induced imbalance between the two layers.^49^ Figure S5B reveals that the bending of the needle increased as the width of the needle increased from 1.0 to 3.0 mm, but it decreased thereafter due to the greater force required to bend a wider needle. Moreover, Figure S5C reveals that at an equal length (8.0 cm) and width (3.0 mm), the bending of the needle decreased as the thickness of both the EDT-incorporated and nonresponsive layers increased from 0.5 to 0.8 mm. This decrease was likely due to the greater force required to bend the thicker EDT-incorporated and nonresponsive layers, which outcompeted the force generated from the swelling-induced imbalance between the two layers. Accordingly, we selected the needle design with a length of 8.0 cm, width of 3.0 mm, and height of 1.0 mm, with both the EDT-incorporated and nonresponsive layers having a thickness of 0.5 mm to maximize the bending of the needle.

**Figure 2:**
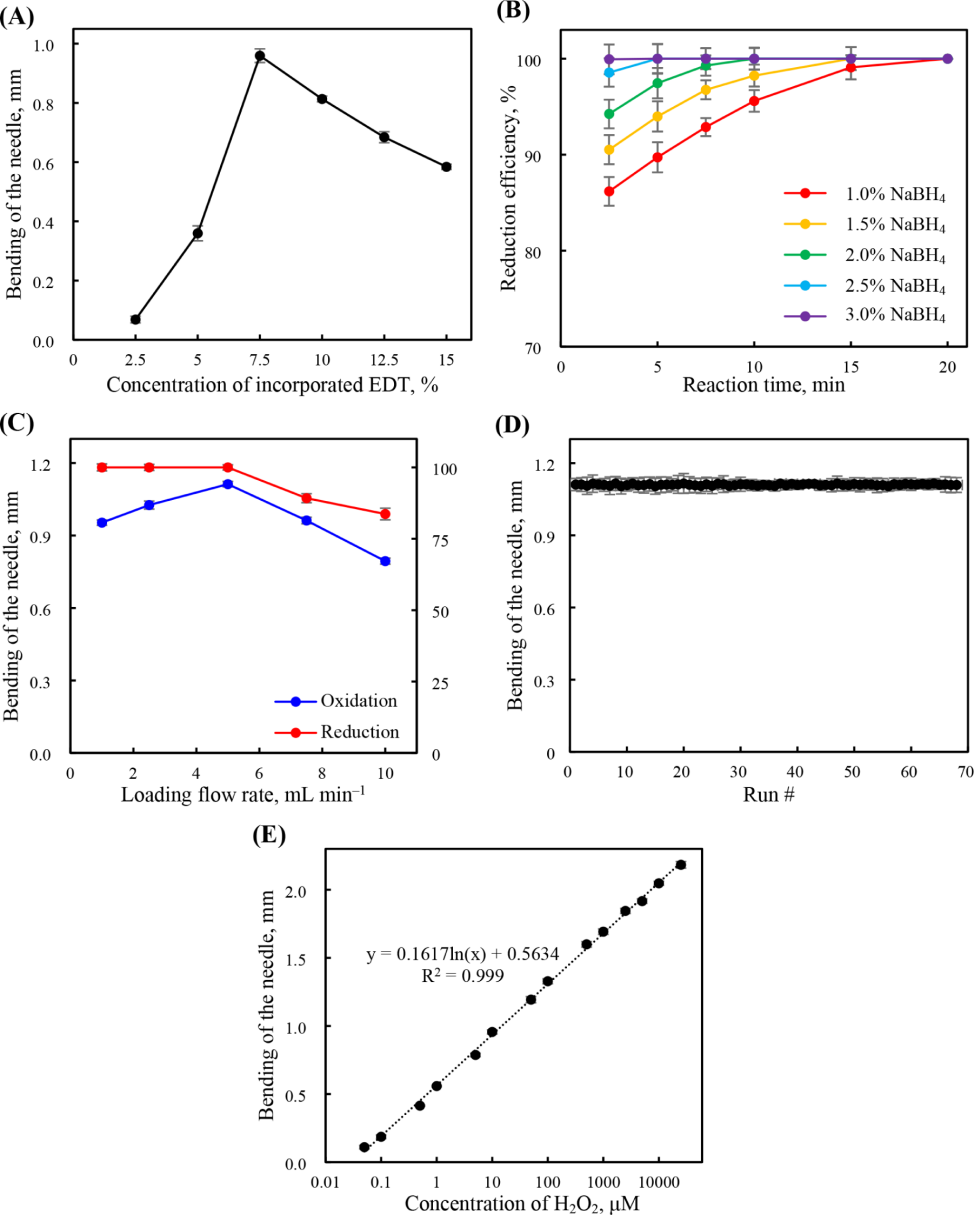
Bending of the needle plotted with respect to the (A) concentration of incorporated EDT, (D) run number, and (E) concentration of H_2_O_2_ (without MD sampling). (B) Reduction efficiency plotted with respect to the NaBH_4_ concentration and reduction time. (C) The effects of the loading flow rate on the bending of the needle and the reduction efficiency. The bending of the needle was measured from the images with analysis using ImageJ software. The reduction efficiency was calculated as the ratio of the recovery of the needle to the bending of the needle. In (A)–(C), the tested solution was 10 μM H_2_O_2_. In (D), the tested solution was 125 μM glucose. Error bars represent the standard deviations (*n* = 8).

After the thioether groups of the copolymer were oxidized to form sulfoxide and sulfone groups, which induced bending of the needle, we used a NaBH_4_ solution to completely reduce these groups and enable the needle to return to its initial position for subsequent glucose and lactate determinations without significant carryover effects.^47^ Figures 2B and S5D show that both the composition of the NaBH_4_ solution and the reduction time significantly influenced the reduction of the sulfoxide and sulfone groups. The reduction efficiencies (represented as the recoveries of the needles) increased with higher concentrations of NaBH_4_ and longer reduction times, reaching their maximum at neutral conditions (pH between 6.0 and 8.0). Since a shorter reduction time resulted in a higher sample throughput, we selected the 2.5% (w/v) NaBH_4_ solution prepared in the 1.0 mM phosphate buffer (pH 8.0) and a reduction time of 5.0 min as the optimal conditions to completely reduce the sulfoxide and sulfone groups, ensuring the recovery of the needle before analyzing the next sample.

Figure 2C illustrates that the loading flow rates of the treated microdialysate (which relate to the dilution of the microdialysate with the carrier stream) and NaBH_4_ solution both influenced the oxidation and reduction of the needle. The maximal bending of the needle occurred at a loading flow rate of 5.0 mL min^–1^, and near 100% reduction efficiencies (needle recovery) were observed when the loading flow rates were in the range from 1.0 to 5.0 mL min^–1^. Based on these findings, we selected 5.0 mL min^–1^ as the optimal flow rate for loading both the treated microdialysate and NaBH_4_ solution into the needle-flow reactors. In addition, we incorporated an evacuation step using an air stream (5.0 mL min^–1^) before loading the NaBH_4_ solution (after measuring the bending of the needle) and a reconditioning step using the 10 mM citrate buffer (pH 6.0; 5.0 mL min^–1^) before loading the next sample (after the recovery of the needle) to prevent the mixing of the oxidase-treated microdialysate and NaBH_4_ solution and developed an online automatic analytical system (Figure S4) that minimized errors from the laboratory environment and manual operation. The relative standard deviation (RSD) of the bending of the needle over 68 continuous redox cycles was 0.3% (Figure 2D; intradevice variations; using the glucose derivatization conditions), demonstrating the excellent reversible shape programming of the 4D-printed needle-flow reactors and their reusability for quantitative chemical analysis without requiring optical spectrometric (chromogenic reagent-based) or fluorescence (fluorogenic reagent-based) determination. Figure 2E reveals a strong correlation between the bending of the needle and H_2_O_2_ concentrations from 5.0 × 10^–2^ to 2.5 × 10^4^ μM (semilogarithmic plot of the bending of the needle versus [H_2_O_2_]; without MD sampling), with a correlation coefficient (*R*) of 0.9990 and method detection limits (MDLs, defined as three times the standard deviation of the bending of the needle from eight blank measurements) of 0.02 μM. These results indicated that the 4D-printed redox-responsive needle-flow reactors were highly suitable for the sensitive determination of H_2_O_2_ based on the induced bending of the needles.

### Online Determination of Glucose and Lactate with 4D-Printed Needle-Flow Reactors

After optimizing the design and fabrication of the needle-flow reactors, we integrated them with GOx- and LOx-mediated derivatization reactions to enable glucose and lactate determination in microdialysates based on the bending of the needle. Since both the redox-responsive properties of the EDT-incorporated copolymer and the efficiencies of the derivatization reactions in generating H_2_O_2_ influenced the bending of the needle, we evaluated the key parameters, including GOx and LOx concentrations, reaction time, buffer composition, pH, temperature, and potential interferences. As shown in Figure 3A,B, the bending of the needle increased with increasing reaction time (ranging from 2.5 to 30 min) and concentrations of GOx and LOx, reaching saturation at 5.0 and 0.01 U mL^–1^ for glucose and lactate, respectively. Considering that a shorter reaction time increased the sample throughput, we set the GOx and LOx concentrations at 5.0 and 0.01 U mL^–1^, respectively, and the reaction time at 5.0 min for online glucose and lactate determination with a stopped-flow step. Figure S5E reveals that the bending of the needle remained consistent across different reaction buffers (acetate, bicarbonate, citrate, maleate, and phosphate, each at 10 mM), with relative differences in the bending of the needles ranging from −0.2% to +0.9% compared to those in the citrate buffer. Figure 3C further indicates that the optimal pH for maximal bending of the needle was 5.0 for GOx-mediated oxidation and 7.0 for LOx-mediated oxidation.^21,50^ We selected the 10 mM citrate buffer (pH 6.0) to allow a single carrier stream to transport both GOx- and LOx-treated microdialysates into their respective needle-flow reactors for measurement. As shown in Figure 3D, the bending of the needle increased with increasing reaction temperature from 20 to 40 °C because of the enhanced derivatization efficiencies.^51,52^ However, beyond 40 °C, the bending decreased because the softer EDT-incorporated copolymer compromised the swelling-induced imbalance between the two layers (Figure S5F).^44^ Consequently, the two needle-flow reactors were thermostated at 40 °C to maximize the induced bending of the needles.

**Figure 3:**
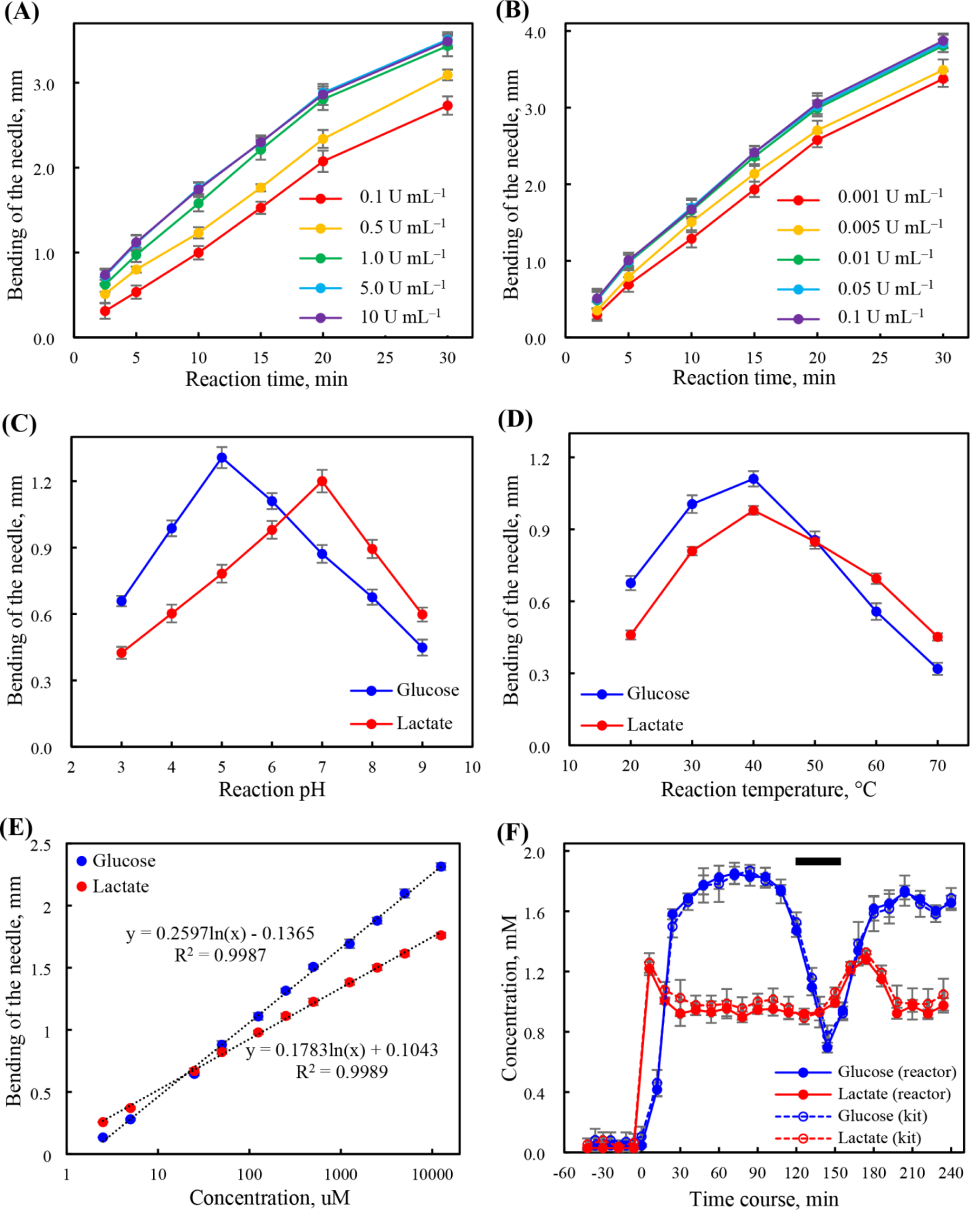
Bending of the needle plotted with respect to the (A) concentration of GOx and reaction time, (B) concentration of LOx and reaction time, (C) reaction pH, (D) reaction temperature, and (E) concentrations of glucose and lactate (calibration curves with MD sampling). (F) Time course of living rat brain extracellular glucose and lactate concentrations following implantation of the MD probe and treatment of perfusion of a high-K^+^ medium. The bending of the needle was measured from the images with the analysis using ImageJ software. In (A)–(D), the tested solutions were 125 μM glucose and lactate. Error bars represent standard deviations (*n* = 8). In (F), error bars represent standard deviations (*n* = 6). Black bar: time interval of perfusing the high-K^+^ medium from the MD probe.

Figure S5G shows that common cations [Na^+^ (10,000 mg L^–1^), K^+^ (10,000 mg L^–1^), Ca^2+^ (1000 mg L^–1^), and Mg^2+^ (1000 mg L^–1^)] and anions [SO_4_^2–^ (10,000 mg L^–1^), F^–^ (1000 mg L^–1^), and Br^–^ (1000 mg L^–1^)] did not affect the bending of the needles, with relative differences ranging from −1.0% to +0.9% compared with those in the citrate buffer in the absence of these ions. This finding indicated that these tested ions had negligible effects on the oxidase-based derivatization efficiencies, the noncovalent interactions between these ions and the sulfoxide and sulfone groups did not significantly inhibit the swelling of the EDT-incorporated layer, and the 4D-printed needle-flow reactors enabled interference-free determination of glucose and lactate in real samples. The RSD of the induced bending of the needle from seven flow reactors was 1.6% (interdevice variation, using glucose derivatization conditions), demonstrating the consistency of the print–pause–print DLP 4DP technique with the EDT-incorporated photocurable resins in fabricating the needle-flow reactors. Also, the spatial factors contributed to minimal fluctuations in the bending of the needle under the stopped-flow condition. Figure S5H illustrates that when preserved in a black plastic bag, these needles retained stable redox-responsive properties for 3 months, with interday fluctuations (RSD) of 0.3%, suggesting the durability of the 4D-printed needle-flow reactors for long-term operation and storage. These results confirmed that the 4D-printed redox-responsive needle-flow reactors exhibited resistance to the buffer and interfering ions, maintained high consistency and durability, and are well-suited for coupling with H_2_O_2_-generating derivatization schemes for online quantitative chemical analysis.

### Analytical Characteristics

When integrating the MD sampling, oxidase-based derivatization schemes, and 4D-printed redox-responsive needle-flow reactors in an online automatic analytical system [sample throughput (temporal resolution): 10 h^–1^ (5 h^–1^ for glucose or lactate alone)], the calibration curves for glucose and lactate followed a semilogarithmic relationship (bending of the needles versus log­[glucose] and log­[lactate]) under optimized conditions (Table S2), with *R* values of 0.9987 and 0.9989, respectively, over a working range of 0.0025 to 12.5 mM (Figure 3E). The sharp changes in the bending of the needle at lower concentrations enabled MDLs of 0.3 μM for glucose and 0.4 μM for lactate. Compared with assay kits and microplate reader measurements, which yielded MDLs of 8.4 and 8.7 μM for glucose and lactate, respectively, our method demonstrated significantly higher sensitivity for glucose and lactate determination. Table S3 shows that the measured glucose and lactate concentrations in the collected samples ranged from 0.030 ± 0.002 to 3.122 ± 0.094 mM and from 0.005 ± 0.0002 to 15.347 ± 0.231 mM, respectively, aligning with reported values^53–57^ and those obtained using glucose and lactate assay kits [relative errors (REs): −1.3% to +4.9% for glucose and −0.7% to +3.8% for lactate; all *p* values >0.4520 (statistical significance level: *p* < 0.05)]. Furthermore, spike recoveries for these samples ranged from 99% to 110% for glucose and 96% to 104% for lactate, confirming the reliability of the 4D-printed redox-responsive needle-flow reactors with oxidase-based derivatization schemes for glucose and lactate determination in real biological samples. Compared with reported optical spectrometric, fluorescence, and electrochemical methods for online glucose and lactate determination (Table S4),^16–23,58–60^ this method offered better operational simplicity (eliminating the need for a spectrophotometer, fluorometer, microplate reader, or electrochemical analyzer), lower MDLs, a broader working range, and reduced cost. In addition to oxidase-based H_2_O_2_-generating derivatization schemes for their substrates (e.g., cholesterol, choline, galactose, glutamate, pyruvate, spermine, and xanthine oxidases), the 4D-printed needle-flow reactors were expected to serve as a reliable and universal sensing component adaptable to other analytical methods involving the generation and/or detection of redox-active substances, including assays based on measuring catalase or peroxidase activities as well as enzyme-linked immunosorbent assay schemes for quantifying antibodies or antigens.

### Online Monitoring of Living Rat Brain Extracellular Glucose and Lactate

We utilized the online automatic analytical system for quantitative profiling of living rat brain extracellular glucose and lactate (*n* = 6), with neuronal depolarization triggered by switching the perfusate from 0.9% NaCl to 1.15% KCl. Figure 3F shows that (i) a relatively high concentration of extracellular lactate was observed during the initial postimplantation period because of local disturbances around the insertion region,^61^ (ii) the basal rat brain extracellular glucose and lactate concentrations were 1.81 ± 0.04 and 0.93 ± 0.02 mM (Figure 1C,D), respectively, between 48 and 120 min postimplantation,^19–21,23,62,63^ (iii) the glucose concentration decreased to 0.70 ± 0.03 mM (39% of the basal value) at 36 min post-treatment (following the perfusate switch), (iv) the lactate concentration increased to 1.28 ± 0.03 mM (137% of the basal value) at 60 min post-treatment,^20,21,23,64,65^ and (v) both glucose and lactate returned to basal levels at 72 min post-treatment for glucose (1.66 ± 0.04 mM) and 96 min post-treatment for lactate (0.95 ± 0.03 mM). When the high content of K^+^ ions diffused abnormally into the brain extracellular fluids, glycolysis dominated the energy support to maintain ion homeostasis by removing excess extracellular K^+^ ions. This process resulted in a decrease in extracellular glucose and a delayed increase in extracellular lactate.^66,67^ The measured basal rat brain extracellular glucose and lactate concentrations were consistent with previously reported values,^19–21,23,62,63^ and the depolarization-induced dynamic profiles aligned with those observed in the same animal model.^20,21,23,64,65^ Moreover, based on the use of glucose and lactate assay kits (with a 250-fold dilution of microdialysates), the measured basal rat brain extracellular glucose and lactate concentrations were 1.80 ± 0.05 and 0.95 ± 0.04 mM, respectively. No significant differences were observed between the 4D-printed needle-flow reactors and assay kits, with *p*-values of 0.7101 for glucose and 0.2990 for lactate. Moreover, the obtained depolarization-induced profiles of rat brain extracellular glucose and lactate from both methods were highly similar (Figure 3F), highlighting the excellent reliability and applicability of our approach for the online quantitative profiling of living rat brain extracellular glucose and lactate.

The massive release of K^+^ ions into the brain’s extracellular fluids profoundly affected the energy metabolism and local synaptic signaling, especially in terms of traumatic brain injury.^1–4,65–69^ From both a biomedical and analytical perspective, tools for online profiling of dynamic variations in brain extracellular glucose and lactate are crucial for studying the kinetics of anaerobic glycolysis in maintaining Na^+^/K^+^ homeostasis. Our method, demonstrated through animal experiments, exhibited sufficient sensitivity and resolving power to profile the dynamic variations in living rat brain extracellular glucose and lactate in response to stimuli. The primary novelty and significance of this research lie in the pioneering use of 4DP technologies to fabricate a reversible redox-responsive device and develop an automatic analytical system for the online determination of glucose and lactate based on the redox-responsive shape programming. This approach eliminated the need for expensive spectrometers and measurement apparatus. In addition, the study demonstrated that combining 4DP technologies, redox-responsive materials, enzymatic derivatization schemes, MD sampling, and online automatic analytical techniques opened new possibilities for enzymatic assay methods. This innovation holds significant potential for advancing the online quantitative profiling of living rat brain extracellular substances with important implications for enabling more biomedical applications.

## Conclusions

We developed an automatic analytical system by integrating the MD sampling apparatus, oxidase-based H_2_O_2_-generating derivatization schemes, and 4D-printed redox-responsive needle-flow reactors for the online quantitative profiling of living rat brain extracellular glucose and lactate. This system offered several advantages over existing glucose and lactate determination methods. First, no molding, bonding, or postprinting treatment was required, as all parts of the needle-flow reactor were directly printed and ready for assembly. This demonstrated the capability of 3DP and 4DP technologies to accelerate the development and evaluation of redox-responsive analytical devices. Second, the measurement principle and operation of the needle-flow reactors were straightforward and user-friendly, allowing them to function as a universal sensing component without the need for an external power supply, thus extending the applicability of analytical methods that detect H_2_O_2_ or use it as an intermediate. Third, coupling the needle-flow reactors with enzymatic derivatization schemes for glucose and lactate determination eliminated the need for expensive apparatus and chromogenic or fluorogenic reagents, making this system a viable alternative to spectrophotometers, fluorometers, and microplate readers. Fourth, the needle-flow reactors were affordable, reusable, and durable, making them suitable for integration in online analytical systems that minimize contaminants and errors from manual operation. Fifth, the analytical method was sensitive, reliable, and independent of reaction buffers and interfering ions, offering superior analytical performance compared with commercial assay kits for glucose and lactate determination in biological samples. Most importantly, this study represented the first use of a 4D-printed reversible redox-responsive device to enable the online quantitative profiling of living rat brain extracellular glucose and lactate depolarization-induced dynamic variations. We believe the concept and design of this 4D-printed redox-responsive device will contribute to the advancement of the 4DP-based fabrication of stimuli-responsive analytical devices and expand the applicability of noninstrumental methods for online quantitative chemical analysis.

## Supplementary Material





## Notes

Infrared spectra of the nonresponsive and EDT-incorporated layers; detailed dimensions and .stl files of the needle-flow reactor; bending of the needle plotted with respect to the length of the needle, width of the needle, thicknesses of the EDT-incorporated and nonresponsive layers, reaction buffer, reaction temperature, interfering ions, and preservation time; reduction efficiency plotted with respect to the pH of the NaBH_4_ solution; photographs and schematic representation of the 4D-printed needle-flow reactors for online determination of glucose and lactate; operating sequence and optimized conditions of the online automatic analytical system employing the 4D-printed needle-flow reactors; analytical characteristics of reported analytical methods for online glucose and lactate determination (PDF)
(ZIP)
