# Supplementary material for: 4D-Printed Redox-Responsive Needle-Flow Reactors Enabling Online Quantitative Profiling of Living Rat Brain Extracellular Lactate and Glucose
Source: Anal Chem. 2025 Jun 24;97(29):15642–50. doi: 10.1021/acs.analchem.5c01036 (PMC12311888; doi:10.1021/acs.analchem.5c01036)
Supplement: Supplementary file 1 [file ac5c01036_si_001.pdf]

Supporting Information

4D-Printed Redox-Responsive Needle-Flow Reactors Enabling Online Quantitative Profiling of Living Rat Brain Extracellular Lactate and Glucose

Hsiao-Chu Chiu and Cheng-Kuan Su*

Department of Chemistry, National Chung Hsing University, Taichung City 402202, Taiwan, R.O.C.

Corresponding Author

Cheng-Kuan Su

E-mail: cksu@nchu.edu.tw

Table of Content

Page S-3	**Figure S1.** Infrared spectra of the nonresponsive and EDT–incorporated (7.5%) layers, recorded using a Fourier transform infrared spectrometer (Tensor 27, Bruker).
Page S-4	**Figure S2.** Detailed dimensions of the flow cell (top and side view), redox-responsive needle, cover (top and side view).
Page S-5	**Figure S3**. Schematic representation of the online automatic analytical system for quantitative profiling of living rat brain extracellular glucose and lactate.
Page S-6	**Figure S4.** Schematic representation of the online automatic analytical system for quantitative profiling of living rat brain extracellular glucose and lactate.
Page S-10	**Figure S5.** Bending of the needle plotted with respect to the length of the needle, width of the needle, thicknesses of the EDT–incorporated and nonresponsive layers, reaction buffer, reaction temperature (without incorporating a derivatization reaction), interfering ions, and preservation time. Reduction efficiency plotted with respect to the pH of the $NaBH_4$ solution.
Page S-12	**Table S1.** Operating sequence of the online automatic analytical system employing the 4D-printed needle-flow reactors
Page S-13	**Table S2.** Optimized conditions for the online automatic analytical system employing the 4D-printed needle-flow reactors
Page S-14	**Table S3.** Analytical results of glucose and lactate in the collected samples
Page S-15	**Table S4.** Analytical characteristics of reported analytical methods for online glucose and lactate determination

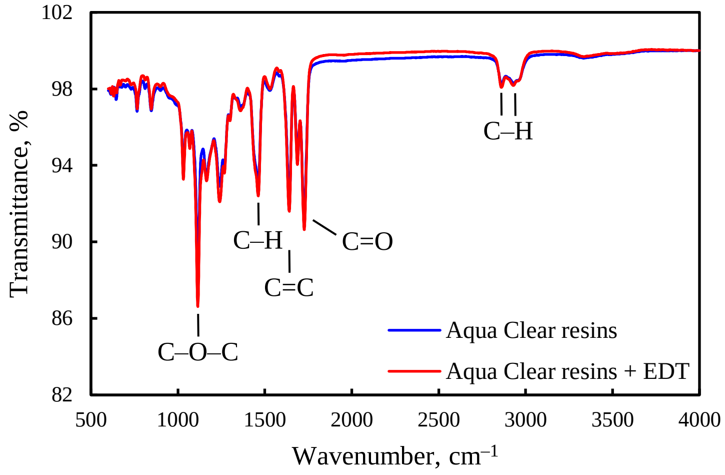

Figure S1. Infrared spectra of the nonresponsive and EDT–incorporated (7.5%) layers, recorded using a Fourier transform infrared spectrometer (Tensor 27, Bruker).

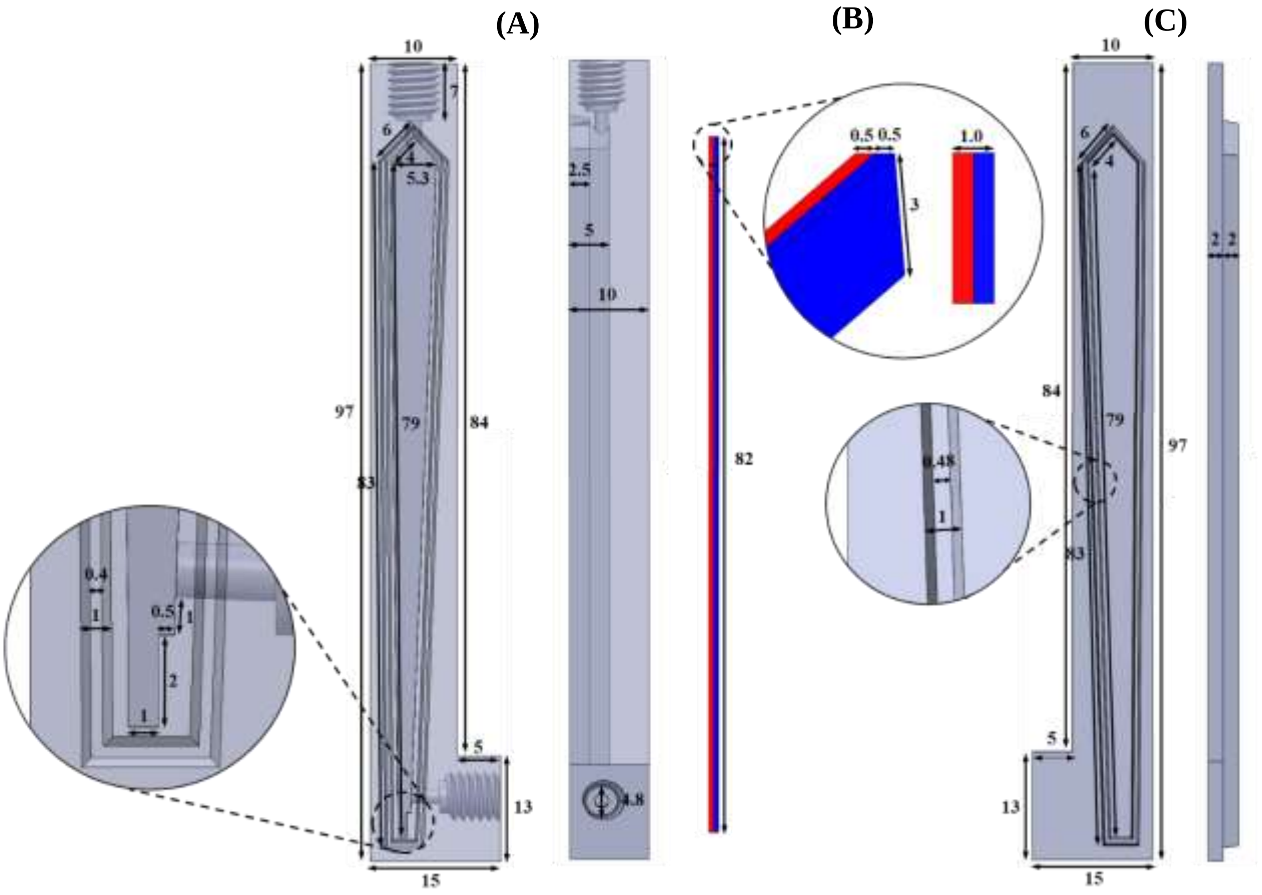

Figure S2. Detailed dimensions of the (A) flow cell (top and side view), (B) redox-responsive needle, (C) cover (top and side view). Units: mm.

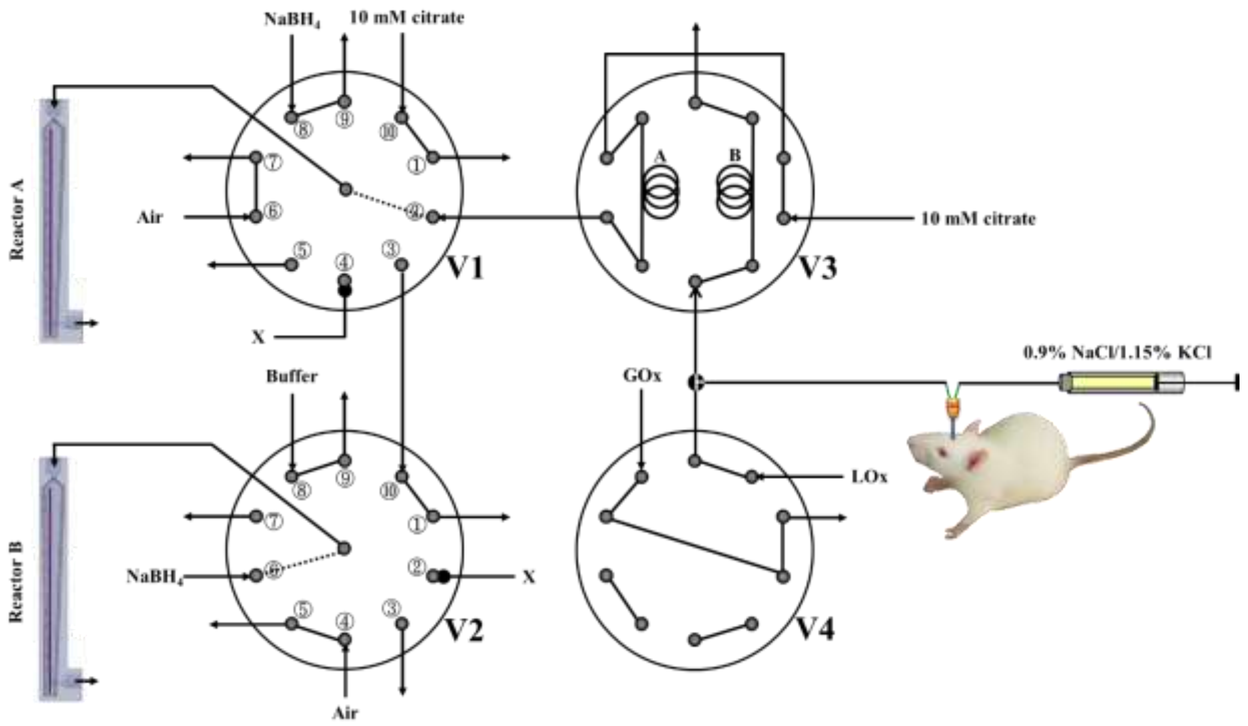

Figure S3. Schematic representation of the online automatic analytical system for quantitative profiling of living rat brain extracellular glucose and lactate. V1 and V2: multi-position, ten-port stream selector; V3 and V4: two-position, ten-port electric switching valve; X: stopped flow with a plug; unmarked arrow: outflow of liquid waste.

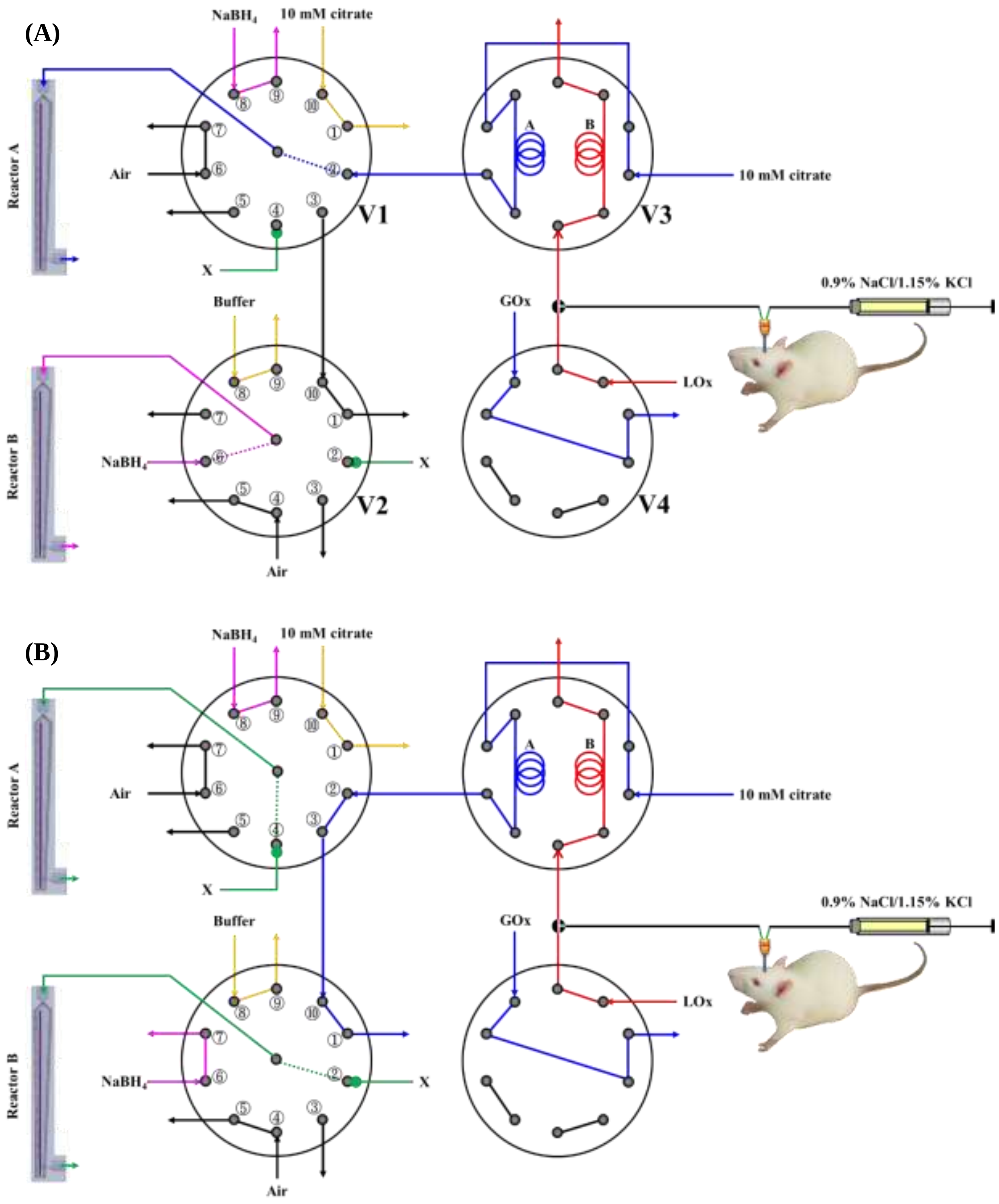
(A)
NaBH4
10 mM citrate
Reactor A
Air
X
V1
V3
A
B
10 mM citrate
Buffer
Reactor B
NaBH4
Air
V2
GOx
LOx
V4
0.9% NaCl/1.15% KCl
(B)
NaBH4
10 mM citrate
Reactor A
Air
X
10 mM citrate
Buffer
Reactor B
NaBH4
Air
GOx
LOx
0.9% NaCl/1.15% KCl

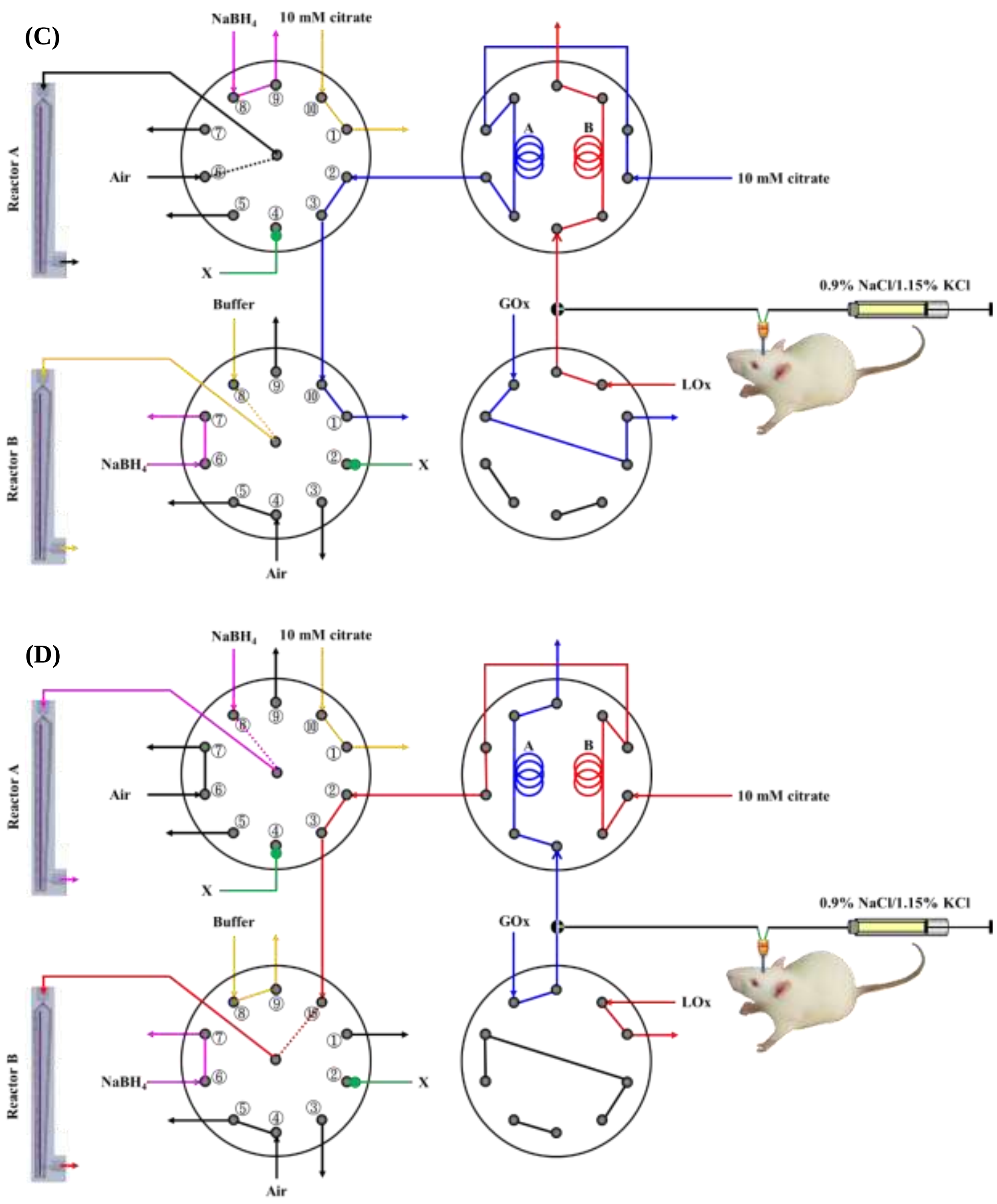

(C)
NaBH4
10 mM citrate
Reactor A
Air
X
A
B
10 mM citrate
Buffer
GOx
0.9% NaCl/1.15% KCl
LOx
Reactor B
NaBH4
X
Air
(D)
NaBH4
10 mM citrate
Reactor A
Air
X
A
B
10 mM citrate
Buffer
GOx
0.9% NaCl/1.15% KCl
LOx
Reactor B
NaBH4
X
Air

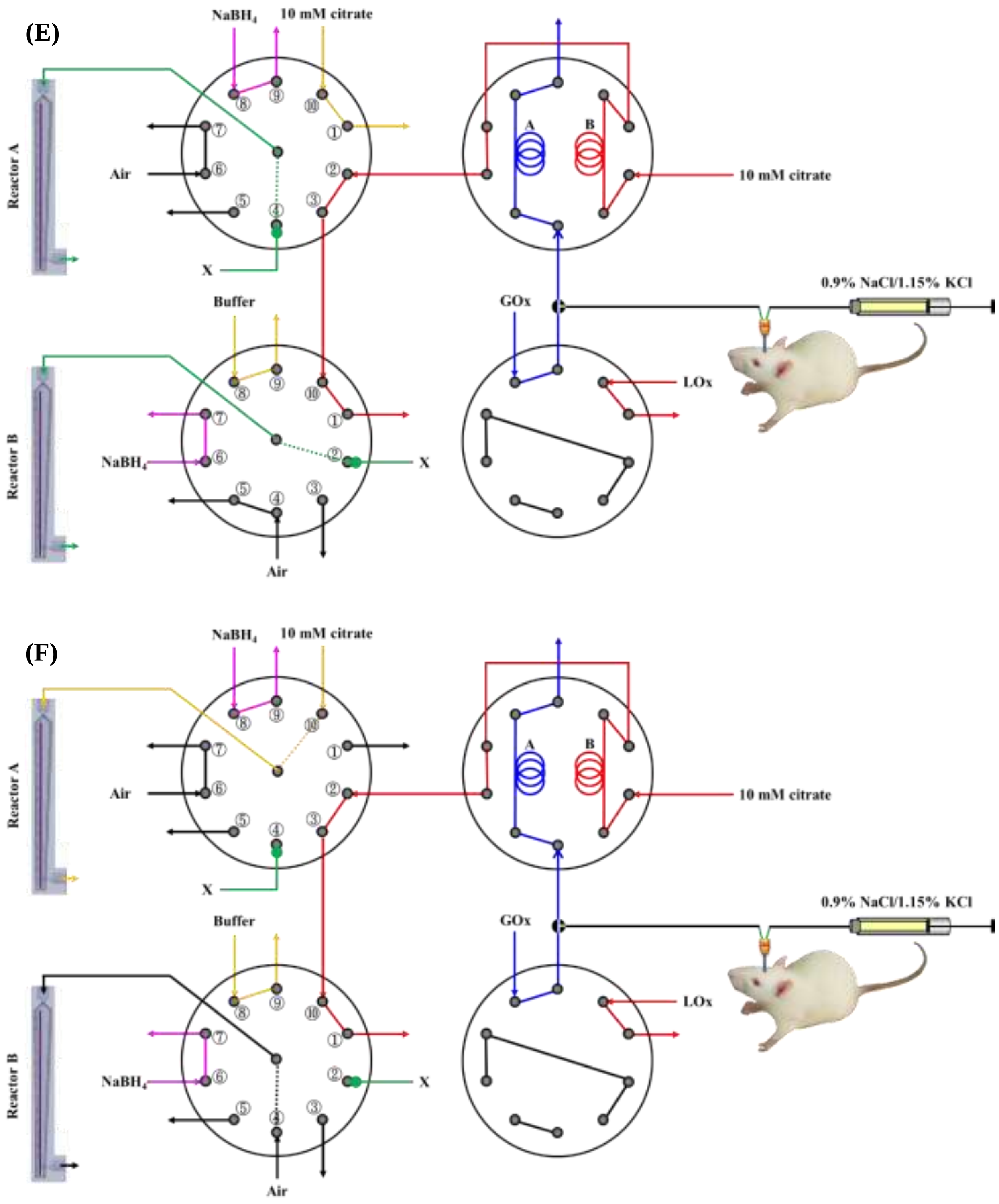

Figure S4. Schematic representation of the online automatic analytical system for quantitative profiling of living rat brain extracellular glucose and lactate. (A) The GOx-treated microdialysate (sample loop A) was loaded into Reactor A using the carrier stream (10 mM citrate buffer, pH 6.0)

while the $NaBH_4$ solution was loaded into Reactor B. (B) The loaded GOx-treated microdialysate induced the bending of the needle in Reactor A for glucose determination while the needle in Reactor B returned to its initial position; both operated under a stopped-flow condition. (C) The loaded microdialysate in Reactor A was evaluated using an air stream which the $NaBH_4$ solution in Reactor B was replaced by 10 mM citrate buffer for loading of a next sample. (D) The LOx-treated microdialysate (sample loop B) was loaded into Reactor B using the carrier stream while the $NaBH_4$ solution was loaded into Reactor A. (E) The loaded LOx-treated microdialysate induced the bending of the needle in Reactor B for lactate determination while the needle in Reactor A returned to its initial position; both operated under a stopped-flow condition. (F) The loaded microdialysate in Reactor B was evaluated using an air stream which the $NaBH_4$ solution in Reactor B was replaced by 10 mM citrate buffer for loading of a next sample. V1 and V2: multi-position, ten-port stream selector; V3 and V4: two-position, ten-port electric switching valve; X: stopped flow with a plug; unmarked arrow: outflow of liquid waste.

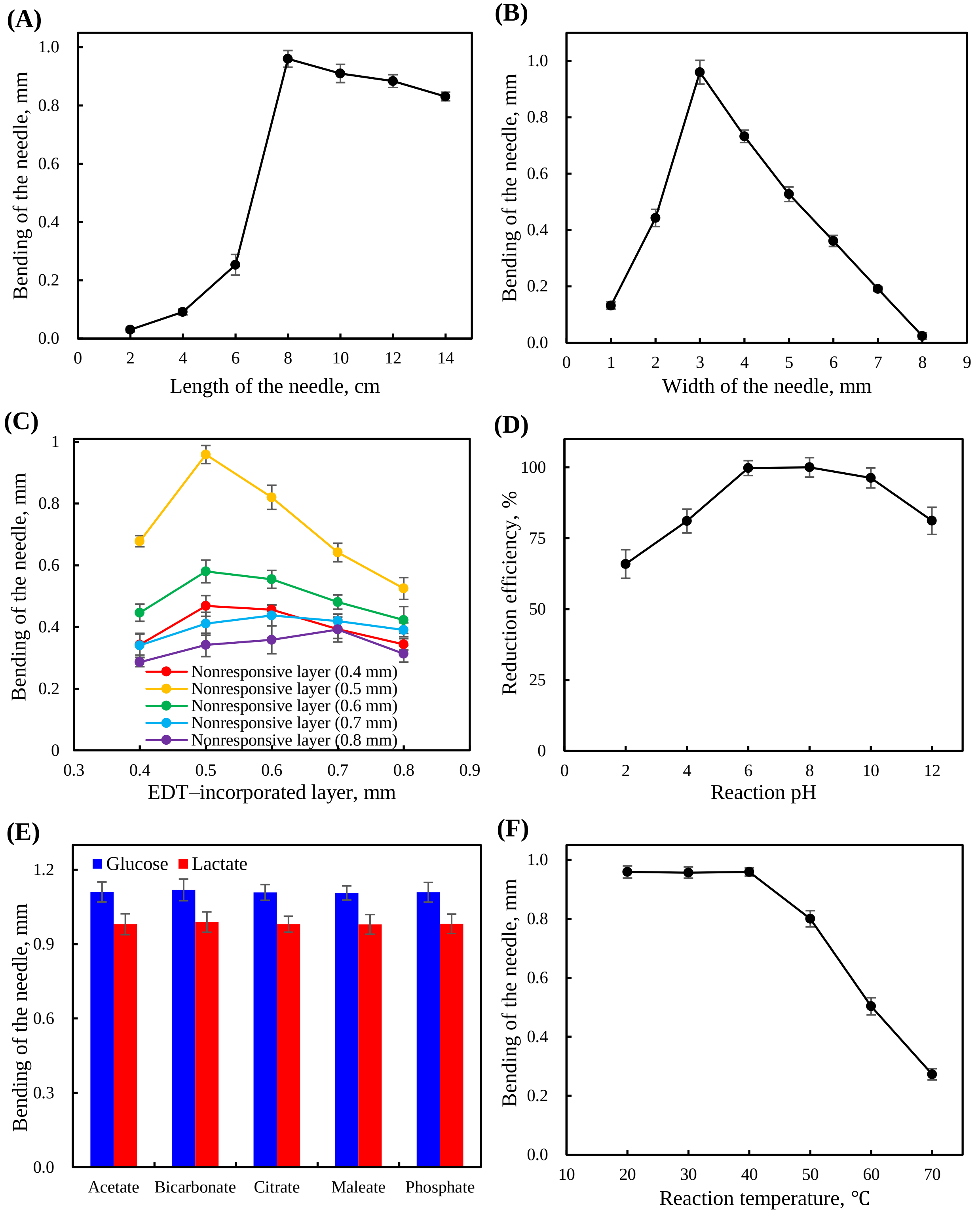

(A)
Bending of the needle, mm
Length of the needle, cm
(B)
Bending of the needle, mm
Width of the needle, mm
(C)
Bending of the needle, mm
EDT–incorporated layer, mm
Nonresponsive layer (0.4 mm)
Nonresponsive layer (0.5 mm)
Nonresponsive layer (0.6 mm)
Nonresponsive layer (0.7 mm)
Nonresponsive layer (0.8 mm)
(D)
Reduction efficiency, %
Reaction pH
(E)
Glucose
Lactate
Bending of the needle, mm
Acetate
Bicarbonate
Citrate
Maleate
Phosphate
(F)
Bending of the needle, mm
Reaction temperature, ℃

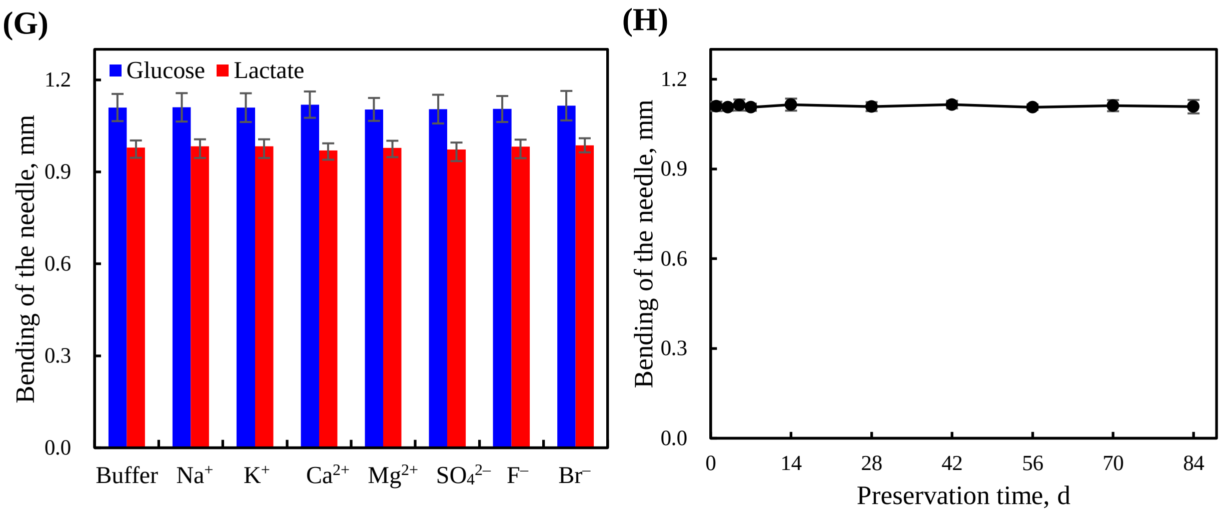

Figure S5. Bending of the needle plotted with respect to the (A) length of the needle, (B) width of the needle, (C) thicknesses of the EDT–incorporated and nonresponsive layers, (E) reaction buffer, (F) reaction temperature (without incorporating a derivatization reaction), (G) interfering ions, and (H) preservation time. (D) Reduction efficiency plotted with respect to the pH of the $NaBH_4$ solution. The bending of the needle was measured from the images with the analysis using ImageJ software. The reduction efficiency was calculated as the ratio of the recovery of the needle to the bending of the needle. In (A)–(D) and (F), the tested solution was 10 μM H_2O_2. In (E) and (G), the tested solutions were 125 μM glucose and lactate. In (H), the tested solution was 125 μM glucose. Error bars represent standard deviations (n = 8).

Table S1. Operating sequence of the online automatic analytical system employing the 4D-printed needle-flow reactors

Step	Valve position (1 → 4)	Time interval	Reactor A	Reactor B
1	2-6-A-A	0:00–0:16	loading of GOx-treated microdialysate	loading of $NaBH_4$ solution
2	4-2-A-A	0:16–5:16	stopped flow for inducing bending of the needle	stopped flow for recovery of the needle
3	6-8-A-A	5:16–6:00	evacuation of sample	evacuation of $NaBH_4$ solution and reconditioning of the needle
4	8-10-B-B	6:00–6:16	loading of $NaBH_4$ solution	loading of LOx-treated microdialysate
5	4-2-B-B	6:16–11:16	stopped flow for recovery of the needle	stopped flow for inducing bending of the needle
6	10-4-B-B	11:16–12:00	evacuation of $NaBH_4$ solution and reconditioning of the needle	evacuation of sample

Table S2. Optimized conditions for the online automatic analytical system employing the 4D-printed needle-flow reactors

**Design and fabrication of the redox-responsive needle**	
Concentration of EDT	7.5%
Length of needle	8.0 cm
Width of needle	3.0 mm
Thickness of needle	1.0 mm
Thickness of EDT–incorporated layer	0.5 mm
Thickness of nonresponsive layer	0.5 mm
**Reduction condition for the redox-responsive needle**	
Reduction solution	2.5% $NaBH_4$ in 1.0 mM phosphate buffer (pH 8.0)
Loading flow rate of reduction solution	5.0 mL min^{-1}
Reduction time	5.0 min
**Online derivatization condition**	
Carrier stream	10 mM citrate buffer, pH 6.0
Loading flow rate of carrier stream	5.0 mL min^{-1}
Concentration of GOx	5.0 U mL^{-1}
Concentration of LOx	0.01 U mL^{-1}
Reaction time and temperature	5.0 min/40 °C
Evacuation medium	air
Evacuation flow rate	5.0 mL min^{-1}
Reconditioning buffer	10 mM citrate buffer, pH 6.0
Reconditioning flow rate	5.0 mL min^{-1}

Table S3. Analytical results of glucose and lactate in the collected samples (n = 8)

Sample	Glucose					Lactate				
	Measured by Reactor, mM	Spike recovery,[a] %	Measured by kit, mM	RE,[b] %	p value	Measured by Reactor, mM	Spike recovery,[a] %	Measured by kit, mM	RE,[b] %	p value
urine 1	0.030 ± 0.002	101	0.029 ± 0.002	+4.9	0.4520	$0.005 \pm 0.000_2$	102	$0.005 \pm 0.000_1$	+3.8	1.0000
urine 2	0.042 ± 0.002	103	0.041 ± 0.003	+2.6	0.5524	$0.006 \pm 0.000_2$	102	$0.006 \pm 0.000_1$	+3.3	1.0000
urine 3	0.051 ± 0.002	102	0.052 ± 0.002	–0.9	0.4520	$0.006 \pm 0.000_2$	97	$0.006 \pm 0.000_2$	+3.3	1.0000
sweat 1	0.166 ± 0.008	100	0.168 ± 0.003	–1.3	0.6149	9.582 ± 0.217	102	9.618 ± 0.097	–0.4	0.7436
sweat 2	0.224 ± 0.008	99	0.223 ± 0.003	+0.4	0.8002	15.347 ± 0.231	98	15.456 ± 0.326	–0.7	0.5588
FBS	3.009 ± 0.120	100	2.988 ± 0.019	+0.7	0.7028	4.513 ± 0.139	96	4.460 ± 0.106	+1.2	0.7763
plasma	3.122 ± 0.094	110	3.140 ± 0.039	–0.6	0.7092	1.383 ± 0.044	104	1.376 ± 0.030	+0.5	0.5169

[a]Spiked concentration: 1 mM.

[b]RE was represented as the difference in the concentrations measured between using the 4D-printed needle-flow reactors and assay kits.

Table S4. Analytical characteristics of reported analytical methods for online glucose and lactate determination

Analytical device/system	Analytical strategy and measurement apparatus	Working range, mM	MDL, μM	sample throughput, h^{-1}	Reference
4D-printed needle-flow reactor	geometric change/smartphone	0.0025–12.5	glucose: 0.3<br>lactate: 0.4	10	this study
online electrochemical system	high-performance liquid chromatography/dual electrochemical detector	0.001–4	0.5	< 3	58
3D-printed copper oxide nanoparticle-functionalized flow reactor	fluorogenic substrate/fluorescence spectrometer	0.05–5	glucose: 6.1	7.5	23
online derivatization system	fluorogenic substrate/luminometer	0–14	glucose: 10	28	16
online electrochemical system	oxidase/prussian blue-based biosensor	0.05–0.5	glucose: 10<br>lactate: 2	--	59
online electrochemical system	nonenzymatic microfluidic sensor	0–16	glucose: 21	--	60
online derivatization system	fluorogenic substrate/luminometer	0–12	glucose: 50	15	17
segmented-flow derivatization system	fluorogenic substrate/fluorescence spectrometer	0.1–2	glucose: 50	> 700	19

sequential enzymatic derivatization system	fluorogenic substrate/fluorescence spectrometer	0.1–10	glucose: 55<br>lactate: 58	6	22
enzyme-immobilized 3D-printed reactor	fluorogenic substrate/fluorescence spectrometer	0.1–5	glucose: 60<br>lactate: 59	15	21
droplet-based micro-flow derivatization system	fluorogenic substrate/luminometer	0.8–10	glucose: 100	20	18
Cu^{2+}-amplified derivatization system	fluorogenic substrate/fluorescence spectrometer	0.1–10	glucose: 180	7.5	20
